# The relationship of adiposity to disease severity in a Crohn's patient cohort

**DOI:** 10.1186/1753-6561-9-S1-A23

**Published:** 2015-01-14

**Authors:** DJ O’Connor, G Sexton, G McCormack

**Affiliations:** 1 School of Medicine, National University of Ireland, Galway, Ireland; 2 Midland Regional Hospital at Tullamore, Arden Road, Tullamore, Co. Offaly, Ireland

## Background

Crohn’s Disease (CD) is a chronic inflammatory bowel disease characterised by recurrent intestinal inflammation [1]. Adipose tissue has metabolic and immune functions regulated through the expression of hormones and cytokines [2,3]. Conventionally, adiposity in CD is believed to reflect disease activity, nutritional status and possibly corticosteroids. Emerging data suggests that adipose tissue may play a more complex immunoregulatory role in CD [4].

## Methods

CD patients attending the gastroenterology department were recruited over a 4 week period were invited to partake in this pilot study. The following data was collected: Extent of disease and previous treatments, current disease activity and biometric measurements of adiposity (Body mass index (BMI), waist hip ratio, mid upper arm circumference, skin fold thickness and percentage body fat using biometric impedance analysis (BIA)).

## Results

27 patients were recruited in this pilot study. 16 (59%) had BMI >25 and (classified as overweight or obese), 10 had normal BMI and 1 had BMI <18. 32% had body fat stores above normal, 44% within normal range and 24% had low fat stores as measured with BIA. Numbers were too small in this pilot study to establish a relationship between disease pattern and/or activity, those requiring >1 course of steroids in the previous year and those on anti-TNF therapy were more likely to have normal range BMI than the group as a whole. Self reported abdominal pain and decreased well being was highest in patients with an increased BMI.

## Conclusions

Obesity rates in the general population are rising [5]. Our study indicates that obesity does present in the CD population. Adipose tissue may be a source of proinflammatory cytokines thus altering the natural history of CD in these patients [6]. Even if there is no impact on disease progression, our findings have important implications for current CD drug and nutritional management.
